# Regional metastasis to anatomies beyond traditional neck dissection boundaries: a multi-institutional analysis focused on unconventional metastases in oral cancer patients

**DOI:** 10.1186/s12957-020-02057-6

**Published:** 2020-10-28

**Authors:** Weijin Gao, Zhuowei Tian, Xiaodan Fang, Jincai Xue, Zhixiang Li, Cong Yang, Chunyue Ma

**Affiliations:** 1 Department of Oral & Maxillofacial–Head & Neck Oncology, 9th People’s Hospital, Shanghai Jiao Tong University School of Medicine, Shanghai Key Laboratory of Stomatology, No. 639, Zhi Zao Ju Road, Shanghai, 200011 China; 2 Department of Oral and Maxillofacial Surgery, The First Affiliated Hospital of Wenzhou Medical University, Wenzhou, 325000 Zhejiang China; 3 Department of Oral and Maxillofacial Surgery, Xiangya Stomatological Hospital, Central South University, Changsha, 410000 Hunan China; 4 Department of Head and Neck Surgery, Gansu Province Tumor Hospital, Lanzhou, 730050 Gansu China; 5 Department of Otolaryngology Head Neck Surgery, The People’s Hospital of Wenshan Prefecture, Wenshan, 663099 Yunnan China

**Keywords:** Sublingual, Buccinators, Parotid, Metastasis, Neck dissections

## Abstract

**Background:**

Regional metastasis sometimes occurs in anatomies that are not included in traditional neck dissections. The purpose of this study was to evaluate the treatment outcomes of squamous cell carcinoma of oral cavity (SCCOC) patients with unconventional metastatic lymph nodes (UMLNs) in sublingual, buccinator, and parotid anatomies.

**Methods:**

This retrospective multi-institutional analysis of squamous cell carcinoma of oral cavity patients with unconventional metastatic lymph nodes was performed from January 2008 to December 2015. All the included patients received surgical treatment for unconventional metastatic lymph nodes. The end point of the study was to determine the factors influencing these patients’ survival and the corresponding solutions to improve survival. Pathological grade, contralateral metastasis, extranodal extension, and other factors were collected and analyzed by logistic regression and the Cox model.

**Results:**

A total of 89 patients were identified. Among these patients, 25 (28.1%) received primary treatment, 28 (31.5%) received staged (therapeutic) neck dissections, and 36 (40.4%) had recurrent or residual diseases. Altogether, 45 patients (51%) had buccinator node metastases, 31 (35%) had sublingual metastases, 12 (14%) had parotid metastases, and 1 had both buccinator and parotid metastases. Regarding regional metastases, 31 patients (34.8%) had isolated unconventional metastatic lymph nodes. Adjuvant therapies were administered to 72 (80.9%) patients, 25 (28.1%) of whom were treated with radio-chemotherapies. The overall survival rate was 38.2%. Multivariate analysis found that the subsites of unconventional metastatic lymph nodes (*P* = 0.029), extranodal extension in both unconventional metastatic lymph nodes (*P* = 0.025) and cervical lymph nodes (*P* = 0.015), sites of primary or recurrent squamous cell carcinoma of oral cavity (*P* = 0.035), and types of neck dissections (*P* = 0.025) were significantly associated with overall survival.

**Conclusions:**

Unconventional metastatic lymph nodes are uncommon, yet awareness of potential unconventional metastatic lymph nodes should be heightened. Early surgical interventions are warranted in patients with sublingual or buccinator metastases, while caution should be given to those with parotid metastases. Aggressive en bloc (in-continuity) resections may be mandatory in advanced oral cancer cases for close anatomic locations with possible buccal or sublingual metastases.

**Supplementary Information:**

The online version contains supplementary material available at 10.1186/s12957-020-02057-6.

## Introduction

Squamous cell carcinoma of the oral cavity (SCCOC) harbors a high likelihood of metastasis due to its aggressive nature and a rich network of interconnected regional lymphatics [1, 2]. The status of cervical lymph nodes is the most important prognostic factor for head and neck cancer, with a reduction of approximately 30–50% in overall survival with the presence of regional metastasis [3]. As recommended in the updated 2019 National Comprehensive Cancer Network (NCCN) guidelines, selective neck dissection is often indicated for the removal of commonly involved lymph nodes found above or extended beyond the omohyoid muscle (levels I–III and sometimes levels I–V) [4]. However, despite the relatively predictable cervical lymph node drainage patterns, SCCOC will sometimes metastasize to regions that are largely unattended or neglected from a conventional viewpoint. Although this type of regional metastasis is occasionally found in advanced cutaneous cancers, it is generally uncommon in SCCOCs [5–7]. These latent unconventional metastatic lymph nodes (UMLNs) are mainly buccinator, sublingual, and parotid lymph nodes, which are not commonly included in traditional neck dissections for SCCOCs [5–10]. The incidence of metastases in these unconventional regions is considered extremely low since most reports are on a single case or small series. However, in our clinical experiences regarding recurrent or advanced SCCOC cases, the presence of these metastases is not as rare as generally considered. For instance, in referred patients with recurrent or advanced squamous cell carcinomas (SCCs) in the upper gingiva or retromolar regions, the presence of buccinator or parotid gland metastasis is not uncommon. For patients with tongue SCCs, sublingual lymph node involvement will sometimes be discovered during the posttreatment follow-up, especially when the sublingual glands or surrounding tissues are left untreated in the first place. Questions have been raised about the treatment regimens for these UMLNs beyond the traditional neck dissection boundaries. In addition, further issues remain unanswered regarding whether the survival outcomes of recurrent SCCOC patients with these additional UMLNs justify aggressive salvage surgeries. Therefore, the purpose of this study was to answer these clinical questions. Our results revealed that the overall survival of SCCOC patients with UMLNs was generally unfavorable. Aggressive and early surgical interventions were needed for patients with UMLNs with or without other adverse clinical factors.

## Methods and materials

**Fig. 1:**
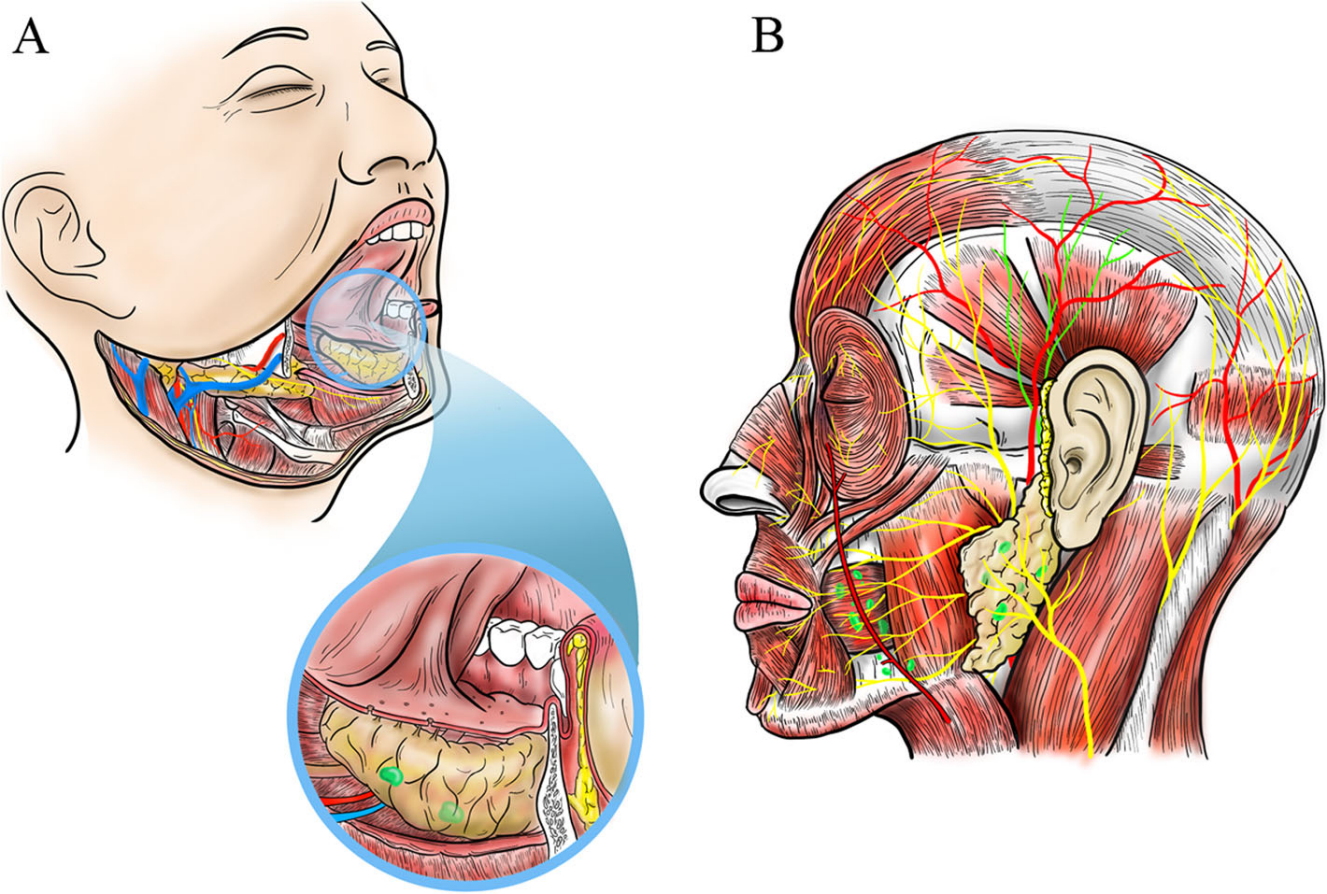
Diagrams of the UMLNs in sublingual, buccinators, or parotid regions for SCCOC. **a** The sublingual lymph nodes lateral (Green) or medial (Light green) to the sublingual gland is depicted. **b** The buccinator and parotid lymph nodes are presented as green nodules

A retrospective review of chart and pathological results was conducted in the databases of four domestic institutions (Shanghai, Changsha, Lanzhou, Wenshan) from January 2008 to December 2015. The follow-up started from the primary operation lasting for 5 years so that the overall survival rate could be counted. Due to the retrospective nature, exemption of ethical approval was granted from the Institutional Ethical Committee. Sublingual lymph nodes were defined as lymph nodes located around the sublingual gland (between the lingual septum and the medial side of the genioglossus muscle or lateral to the genioglossus muscle). Buccinator (or facial) lymph nodes were defined as lymph nodes above the lower mandible ridge and confined to the buccinator area. Parotid lymph nodes included both superficial- or deep-lobe parotid tissues, with the exclusion of parotid tails (Fig. 1, supplementary figures). The inclusion criteria were as follows: (1) patients with primary or recurrent SCCOCs or with secondary therapeutic neck dissections after failed watchful observations; (2) patients with pathologically confirmed metastases in sublingual, buccinator, or parotid lymph nodes; (3) patients with metastases with a distance of at least 2 cm from the primary or recurrent lesions and without direct mucosal ulcerated presentations (for blurred distinctions from recurrences or secondary primaries); (4) patients treated with curative rather than palliative intent; and (5) patients with recurrent lesions with an initial history of negative surgical margins. The exclusion criteria were as follows: (1) patients with buccal SCCs and metastases in ipsilateral buccinator lymph nodes were excluded for ambiguity between metastases or direct invasions by primary or recurrent lesions; (2) patients with floor of mouth SCCs and metastases in sublingual lymph nodes were excluded for much the same reason; and (3) patients with primary or recurrent cutaneous lesions (skin cancers). All the patients included in this study received surgeries and/or other adjuvant therapies for disease management.

Clinical and demographic characteristics were collected and analyzed for an overview of the study population. The basic information included, but was not limited to, the patient’s age, sex, and histories of smoking/alcohol or premalignant mucosal diseases (leukoplakia and lichen planus). A history of prior treatment was recorded as well. In addition, the subsites of the UMLNs were collected. To assess patients’ locoregional conditions, not only the stages for primary lesions (based on the 8th edition of the American Joint Committee on Cancer (AJCC) staging system [11]) but also other detailed pathological data (such as pathological upgrades (prior vs. present), depth of invasion, perineural invasion (PNI), and extra-nodal extensions (ENE)) were reviewed. Though undefined at traditional levels, UMLNs were also counted as cervical metastases to avoid underestimation of their regional aggressiveness. In addition, treatment information regarding neck dissections was obtained for additional metastatic conditions other than UMLNs. According to the American Head and Neck Society, all neck dissections in this study should be classified as “extended neck dissections” due to the involvement of UMLN structures [12]. However, for the sake of comparison, we tended to classify specific types of neck dissections in terms of the coverage of traditional cervical levels, such as “ipsilateral selective neck dissection (ISND) (levels I to III),” “ipsilateral radical neck dissection (IRND) (levels I to V),” “bilateral neck dissection (BND),” and “contralateral neck dissection (CND).” On the other hand, sublingual, buccinator, and parotid lymph nodes, which were the focus of this study, were independently analyzed with detailed statistics, such as sizes and ENE.

Because lymphatic drainage after treatment may change, according to different admission statuses in our study, the whole population was thus divided into 3 groups: those with primary lesions (PG); those with staged (secondary) neck dissections (SG) with a primary oral cancer resection previously but without neck dissection; and those with recurrent or residual lesions for salvage surgery (RRG) with neck dissection before but with developed clinical metastasis later. Notably, due to the anatomic vicinity of UMLNs and primary SCCOCs, we subclassified those with primary tongue, buccal, floor-of-mouth cancers as the “suspected residual group”, while the others were regarded as “true recurrent group.” In addition, those with both tongue cancers and sublingual node metastases were investigated for the treatment implications and prognostic values.

Information regarding prior and postoperative radio- and chemotherapies was also presented. For prior radiation histories, buccinator regions were generally not included in the prior radiation target fields unless with direct disease involvement, while sublingual glands were included in the prior radiation clinical target volumes for oral cancers involving the tongue, floor of mouth, and lower and retromolar gingiva out of concern for close margin distances. In addition, the ipsilateral parotid glands were always included in prior radiation fields.

For targeted therapies, only a few patients with very advanced disease received adjuvant anti-epithelial growth factor receptor (EGFR) treatment. With the purposes of this study in mind, questions about the clinical and prognostic discrepancies between these groups were attempted. All patients were routinely followed up. Information with respect to deaths was also collected.

The statistical analyses were performed with SPSS version 23.0 software (IBM Corp., Armonk, NY). Logistic regression was utilized to determine the correlated factors. Survival curves were generated by the Kaplan-Meier method. Additionally, the multivariate proportional hazard Cox model was used to evaluate the prognostic factors.

## Results

In total, among 6357 oral cancer patients from these 4 institutions who had received neck dissections, 89 patients (54 male and 35 female) had UMLNs (the mean age was 57, range 31–87). The incidence of UMLNs was approximately 1.4% in this study. The follow-up period ranged from 2 to 117 months (mean 40.4 months). Primary or recurrent sites included the lip, buccal mucosa, upper gingiva, lower gingiva, floor of mouth, tongue, retromolar trigone, and hard palate. Premalignant mucosal diseases were found in 27 (25.8%) patients, while 49 patients had histories of smoking or alcohol. Among all the patients, 25 (28.1%) were admitted for primary treatment, 28 (31.5%) for staged (therapeutic) neck dissections alone, and 36 (40.4%) for recurrent diseases. Within those admitted for primary treatment, only 6 (out of 25) patients had T1-2 lesions. A detailed description of the patients’ general clinicopathologic characteristics is presented in Table 1.

**Table 1:** Patient demographics and tumor characteristics

Variables	***N***(%)	The overall survival rate	Univariate analysis
**Age**			**0.086**
31–59	**37(41.6)**	**43.2**	
60–87	**52(58.4)**	**34.6**	
**Gender**			**0.500**
Male	**54(60.7)**	**37.0**	
Female	**35(39.3)**	**40.0**	
**Histories of smoking and alcohol**			**0.867**
Yes	**49(55.1)**	**55.1**	
No	**40(44.9)**	**35.0**	
**Premalignant mucosal diseases**			**0.257**
Yes	**27(30.3)**	**30.3**	
No	**62(69.7)**	**35.5**	
**Prior radio- or chemo-therapies**			**0.011**
Yes	**22(24.7)**	**24.7**	
No	**67(75.3)**	**43.3**	
**Treatment status**			**< 0.001**
Primary lesions for treatment (PG)	**25(28.1)**	**56.0**	
Staged (secondary) neck dissections (SG)	**28(31.5)**	**53.6**	
Recurrent or residual lesions for salvage surgery (RRG)	**36(43.8)**	**13.9**	
**Primary or recurrent site**			**0.005**
Buccal mucosa	**7(7.9)**	**42.9**	
Upper gingiva	**13(14.6)**	**30.8**	
Lower gingiva	**12(13.5)**	**41.7**	
Floor of mouth	**5(5.6)**	**20.0**	
Tongue	**24(27.0)**	**37.5**	
Retromolar trigone	**9(10.1)**	**0.0**	
Lip	**6(6.7)**	**83.3**	
Hard palate	**13(14.6)**	**53.8**	
**Unconventional metastatic subsites**			**0.008**
Buccinator	**45(50.6)**	**44.4**	
Sublingual	**31(34.8)**	**38.7**	
Parotid^a^	**13(14.6)**	**15.4**	
**Pathological grade**			**0.002**
I	**4(4.5)**	**50.0**	
II	**55(61.8)**	**49.1**	
III	**30(33.7)**	**16.7**	
**Number of metastatic lymph nodes in the unconventional subsites**			**0.087**
1	**68(76.4)**	**42.6**	
2	**20(22.5)**	**25.0**	
3	**1(1.1)**	**0.0**	
**Lymph nodes size of UMLNs**			**0.003**
0–3 cm	**65(73.0)**	**44.6**	
> 3 cm	**24(27.0)**	**20.8**	
**Extranodal extension (ENE) in UMLNs**			**< 0.001**
Yes	**28(31.5)**	**17.9**	
No	**61(68.5)**	**47.5**	
**Contralateral metastasis**			**< 0.001**
Yes	**27(30.3)**	**30.3**	
No	**62(69.7)**	**50.0**	
**Concurrent with cervical lymph node metastases**			**0.003**
Yes	**58(65.2)**	**65.2**	
No	**31(34.8)**	**64.5**	
**Extranodal extension (ENE) in cervical lymph nodes**			**< 0.001**
Yes	**21(23.6)**	**23.6**	
No	**68(76.4)**	**48.5**	
**Neck dissection**			**< 0.001**
ISND	**18(20.2)**	**61.1**	
IRND	**31(34.8)**	**54.8**	
CND	**8(9.0)**	**0.0**	
BND	**25(28.1)**	**12.0**	
None	**7(7.9)**	**42.9**	
**Anti-EGFR therapy**			**0.957**
Yes	**16(18.0)**	**31.3**	
No	**73(82.0)**	**39.7**	
**Postoperative adjuvant therapies**			**0.042**
Radiotherapy	**41(46.1)**	**46.3**	
Chemotherapy	**3(3.4)**	**0.0**	
Radio-chemotherapies	**25(28.1)**	**32.0**	
None	**20(22.5)**	**35.0**	

*NA* not available

^a^Including a case which is with both buccinator and parotid metastases,but mostly in the parotid

As the study focus, specific types of neck dissections were analyzed and compared for regional control. Within the entire study population for current neck dissections, 31 (34.8%) patients received IRNDs, while 25 (28.1%) received BNDs. Among the rest, 18 patients received ISNDs, 8 received CNDs, and 7 did not receive any neck dissections. Among these patients, 27 (30.3%) had ipsilateral level I/II/III metastases, 7 (7.9%) had ipsilateral level IV/V metastases, 24 (27.0%) had contralateral metastases, and the rest had no other metastases. Among the 36 patients in the RRG group, histories of prior ISNDs were found in 13 (36.1%), while 12 (33.3%) had prior IRNDs and 1 (2.8%) had a prior BND. The rest had not previously received neck dissections. For the UMLNs, 45 patients (51%) had buccinator nodal metastases, 31 (35%) had sublingual metastases, 12 (14%) had parotid metastases, and 1 had both buccinator and parotid metastases (representative cases are presented in Supplementary Figures 3, 4, 5, 6, 7, 8 and 9). Most patients (*n* = 63, 70.8%) were confirmed with single positive UMLNs; only 24 (26.9%) patients were found with UMLNs measuring beyond 3 cm in diameter. Apart from the sizes, there were 27 (30.3%) UMLNs with evidence of ENE; 9 patients were further confirmed with ENE in both UMLNs and other lymph nodes of traditional levels.

In regard to adjuvant therapies, 22 patients (24.7%) were found with histories of prior radio- or chemotherapies. On the other hand, postoperative regimens (radio-, chemo-, and/or targeted therapies) were administered to 72 (80.9%) patients, of whom 25 (28.1%) received radio-chemotherapies. A closer analysis revealed that 11 (12.4%) patients received re-irradiation due to locoregional recurrences. Additionally, targeted anti-EGFR therapies were used in 16 (18.0%) patients with very advanced disease, of whom most failed the salvage treatment.

Regarding the causes of death, recurrences were found in the majority of patients (70.9%), of whom 26 (29.2%) had regional (lymphatic relevant) failures, irrespective of local or distant status. Three (3.4%) patients died from causes other than SCCOCs. The overall survival rate reached 38.2%, while the disease-specific survival rate was 41.6%.

Among all these factors, the subsite of UMLNs (*P* = 0.029) and primary or recurrent site of SCCOCs (*P* = 0.035) were significantly associated with overall survival based on the Cox multivariate analysis. In addition, ENE of UMLNs (*P* = 0.025), ENE of cervical lymph nodes (*P* = 0.015), and neck dissection types (*P* = 0.025) were also found to be significantly related to prognosis (Table 2).

**Table 2:** The prognostic factors in the COX model

Variables	COX		
	OR(95%CI)	Wald	***P*** value
**Primary or recurrent site**^a^		**15.113**	**0.035**
Buccal mucosa			
Upper gingiva			
Lower gingiva			
Floor of mouth			
Tongue			
Retromolar trigone			
Lip			
Hard palate			
**Unconventional metastatic subsites**^a^		**7.103**	**0.029**
Buccinator			
Sublingual			
Parotid			
**Pathological grade**	**NA**	**NA**	**0.23**
I			
II			
III			
**Lymph nodes size of UMLNs**	**NA**	**NA**	**0.432**
0–3 cm			
> 3 cm			
**Extranodal extension (ENE) in UMLNs**	**1.17-10.19**	**5.004**	**0.025**
Yes			
No			
**Contralateral metastasis**	**NA**	**NA**	**0.076**
Yes			
No			
**Concurrent with cervical lymph node metastases**	**NA**	**NA**	**0.407**
Yes			
No			
**Extranodal extension (ENE) in cervical lymph nodes**	**1.23-6.81**	**5.909**	**0.015**
Yes			
No			
**Neck dissection**^a^		**11.163**	**0.025**
ISND			
IRND			
CND			
BND			
None			
**Postoperative adjuvant therapies**^a^		**NA**	**0.863**
Radiotherapy			
Chemotherapy			
Radio-chemotherapies			
None			

*NA* not available

-2 Log likelihood = 358.102(*P* < 0.001)

^a^Dummy variable was introduced when analyzing these factors

For the various admission statuses, significant survival differences were found among the PG, SG, and RRG groups (*P* < 0.001). Specifically, only ENE in cervical lymph nodes (*P* = 0.006) and DOI (*P* = 0.021) were statistically significant in the PG group by Cox regression. ENE in cervical lymph nodes was also found to be statistically significant in the RRG group. However, in the SG group, only the types of neck dissections (*P* = 0.001) and ENE of UMLNs (*P* = 0.017) were found to be statistically significant in both multivariate Cox models (Tables 3, 4, and 5, supplementary figures). With a closer investigation of the subgroups of RRG, we found no statistical significance between the “suspected residual group” and the “true recurrent group” (*P* = 0.351). Nevertheless, within the suspected residual group, we found size of metastatic sublingual node (*P* = 0.001), ENE in sublingual node (*P* = 0.001), and the other four parameters reached statistical significance in the Kaplan-Meier methods. Furthermore, ENE in sublingual node (*P* = 0.003) was also found significant in the multivariate analysis, indicatively of a poor prognosis in cases with dual adverse factors of residual disease and nearby UMLN (Supplementary Table 1).

**Table 3:** The prognostic factors for unconventional metastatic patients in the PG group

Variables	The overall survival rate	p Value	
		Univariate Analysis	Cox Model^a^
Age		0.347	NA
31–59(11)	45.5%		
60–87(14)	64.3%		
Gender		0.968	NA
Male(13)	53.8%		
Female(12)	58.3%		
Histories of smoking or alcohol		0.617	NA
Yes(13)	61.5%		
No(12)	50.0%		
Premalignant mucosal diseases		0.957	NA
Yes(12)	58.3%		
No(13)	53.8%		
Primary site		0.251	NA
Buccal mucosa(1)	None		
Upper gingiva(1)	None		
Lower gingiva(5)	80.0%		
Tongue(11)	54.5%		
Carcinoma of lip(4)	33.3%		
Retromolar trigone(1)	None		
Hard palate(2)	50.0%		
Unconventional metastatic subsites		0.183	NA
Buccinator (10)	60.0%		
Sublingual(12)	66.7%		
Parotid(3)	None		
Pathological grade		0.009	NA
II(17)	29.4%		
III(8)	25.0%		
TNM staging		0.197	NA
T1/T2(6)	100.0%		
T3/T4(19)	42.1%		
Number of metastatic lymph nodes in the unconventional sites		0.037	NA
1(19)	68.4%		
2(6)	16.7%		
Lymph nodes size of UMLNs		0.041	NA
0–3 cm(22)	63.6%		
> 3 cm(3)	None		
Extranodal extension (ENE) in UMLNs		0.013	NA
Yes(4)	None		
No(21)	66.7%		
Contralateral metastasis		0.019	NA
Yes(7)	28.6%		
No(18)	66.7%		
Cervical lymph node metastasis		0.197	NA
Yes(19)	42.1%		
No(6)	100.0%		
Neck dissections		0.010	NA
ISND(9)	77.8%		
IRND(8)	62.5%		
BND(8)	25.0%		
Extranodal extension (ENE) in cervical lymph nodes		0.002	0.006
Yes(8)	12.5%		
No(17)	76.5%		
Postoperative adjuvant therapies		0.470	NA
Radiotherapy(13)	53.8%		
Radio-chemotherapies(8)	37.5%		
None(4)	16.7%		
sDepth of invasion			
> 10 mm(12)	25%	0.014	0.021
< 10 mm(13)	84.6%		
Perineural invasion (PNI)			
Yes(9)	22.2%	0.016	NA
No(16)	75.0%		

*NA* not available

^a^Cox analysis for PG group only

**Table 4:** The prognostic factors for unconventional metastatic patients in the SG group

Variables	The overall survival rate	*P* value	
		Univariate analysis	Cox model^a^
Age		0.347	NA
33–59(16)	56.3%		
60–87(12)	50.0%		
Gender		0.541	NA
Male(17)	58.8%		
Female(11)	45.5%		
Histories of smoking or alcohol		0.363	NA
Yes(16)	62.5%		
No(12)	41.7%		
Premalignant mucosal diseases		0.530	NA
Yes(7)	57.1%		
No(21)	52.4%		
Primary site		0.678	NA
Buccal mucosa(1)	100.0%		
Carcinoma of lip(2)	100.0%		
Upper gingiva(5)	60%		
Lower gingiva(2)	None		
Floor of mouth(2)	None		
Tongue(6)	50.0%		
Hard palate(10)	60.0%		
Unconventional metastatic subsites		0.810	NA
Buccinator (19)	57.9%		
Sublingual(8)	37.5%		
Parotid(1)	100%		
Pathological grade		0.890	NA
I(4)	50.0%		
II(19)	52.6%		
III(5)	60.0%		
Pathological grade is upgraded compared to the last surgery		0.929	NA
Yes(5)	60.0%		
No(23)	52.2%		
Number of metastatic lymph nodes in the unconventional subsites		0.344	NA
1(20)	60.0%		
2(8)	37.5%		
Lymph nodes size of UMLNs		0.026	NA
0–3 cm(21)	61.9%		
> 3 cm(7)	28.6%		
Extranodal extension (ENE) in UMLNs		0.015	0.017
Yes(8)	25.0%		
No(20)	65.0%		
Contralateral metastasis		0.006	NA
Yes(7)	14.3%		
No(21)	66.7%		
Cervical lymph node metastasis		0.046	NA
Yes(17)	35.3%		
No(11)	81.8%		
Neck dissections		0.018	0.001
ISND(4)	75.0%		
IRND(16)	68.8%		
BND(8)	12.5%		
Extranodal extension (ENE) in cervical lymph nodes		0.025	NA
Yes(3)	None		
No(25)	60.0%		
Postoperative adjuvant therapies		0.100	NA
Radiotherapy(15)	66.7%		
Radio-chemotherapies(9)	44.4%		
None(4)	25.0%		

*NA* not available

^a^Cox analysis for SG group only

**Table 5:** The prognostic factors for unconventional metastatic patients in the RRG group

Variables	The overall survival rate	*P* value	
		Univariate analysis	Cox model^b^
Age		0.500	NA
33–59(25)	16.0%		
60–87(11)	9.1%		
Gender		0.286	NA
Male(24)	12.5%		
Female(12)	16.7%		
Histories of smoking and alcohol	10.0%	0.248	NA
Yes(20)	10.0%		
No(16)	12.5%		
Premalignant mucosal diseases		0.484	NA
Yes(8)	12.5%		
No(28)	14.3%		
Prior radio- or chemo-therapies		0.809	NA
Yes(21)	19.0%		
No(15)	6.7%		
Primary site		0.838	NA
Buccal mucosa(5)	40.0%		
Upper gingiva(7)	14.3%		
Lower gingiva(5)	20.0%		
Floor of mouth(3)	33.3%		
Retromolar trigone(8)	None		
Tongue(7)	None		
Hard palate(1)	None		
Unconventional metastatic subsites		0.122	NA
Buccinator (16)	18.8%		
Sublingual(11)	9.1%		
Parotid^a^(9)	11.1%		
Pathological grade		0.172	NA
II(19)	26.3%		
III(17)	None		
Pathological grade is upgraded compared to the last surgery		0.404	NA
Yes(16)	6.3%		
No(20)	20.0%		
Number of metastatic lymph nodes in the unconventional subsites		0.617	NA
1(29)	13.8%		
2(6)	16.7%		
3(1)	None		
Lymph nodes size of UMLNs		0.808	NA
0–3 cm(22)	9.1%		
> 3 cm(14)	21.4%		
Last neck dissections		0.407	NA
ISND(13)	7.7%		
IRND(12)	25.0%		
BND(1)	None		
No treatment(10)	10.0%		
Neck dissections this time		0.426	NA
ISND(5)	20.0%		
IRND(7)	14.3%		
CND(8)	None		
BND(9)	None		
No treatment(7)	42.9%		
Extranodal extension (ENE) in UMLNs		0.290	NA
Yes(16)	18.8%		
No(20)	10.0%		
Contralateral metastasis		0.124	NA
Yes(13)	None		
No(23)	21.7%		
Cervical lymph node metastasis		0.066	NA
Yes(22)	None		
No(14)	35.7%		
Extranodal extension (ENE) in cervical lymph nodes		0.030	0.030
Yes(10)	None		
No(26)	19.2%		
Postoperative adjuvant therapies		0.719	NA
Radiotherapy(13)	15.4%		
Chemotherapy(3)	None		
Radio-chemotherapies(8)	12.5%		
None(12)	16.7%		

*NA* not available

^a^Including a case which is with both buccinator and parotid metastases,but mostly in the parotid

^b^Cox analysis for RRG group only

It is no surprise that the locations of primary or recurrent SCCOCs were statistically related to the UMLN subsites, especially for those invading the buccal mucosa (*P* < 0.001), tongue (*P* = 0.024), upper gingiva (*P* = 0.007), retromolar trigone (*P* = 0.018), and lip (*P* = 0.002) (Table 6). And it is found that to tongue cancer patients with sublingual nodes metastasis, treatment status (*P* = 0.003) and pathological grade (*P* = 0.001) could affect the prognostic effects (Supplementary Table 2). We also found that patients with sublingual UMLNs were at increased risk of simultaneous contralateral metastasis (*P* = 0.010). Within these cases, we have noticed that the UMLN of those with tongue cancers were mostly sublingual node metastases (91.7%). This trend of tongue cancers towards sublingual node metastases was also demonstrated by the significant relations between tongue SCCOCs with unconventional metastatic subsites (*P* = 0.024), especially sublingual ones. Within these special tongue SCCOC patients, the parameters, such as treatment status (*P* = 0.003) and pathological grade (*P* = 0.001), could affect the survival outcome (Supplementary Table 2). Meanwhile, patients with parotid UMLNs were more likely to have ENE in UMLNs (*P* = 0.016) and higher pathological grades (*P* = 0.006) (Table 6).

**Table 6:** Factors related to the various locations of SCCOCs

Value A	Value B	***P*** values
**Buccal mucosa**	**Unconventional metastatic subsites**	**< 0.001**
**Tongue**	**Unconventional metastatic subsites**	**0.024**
	**Number of metastatic lymph nodes**	**0.002**
**Floor of mouth**	None*	
**Upper gingiva**	**Histories of smoking or alcohol**	**0.013**
	**Unconventional metastatic subsites**	**0.007**
	**ENE in UMLNs**	**0.029**
	**ENE in cervical lymph nodes**	**0.025**
**Retromolar trigone**	**Unconventional metastatic subsites**	**0.018**
**Lip**	**Unconventional metastatic subsites**	**0.002**
	**Gender**	**0.013**
**Hard palate**	None*	
**Lower gingiva**	None*	

*All the factors were proved no significance (*P* > 0.05)

## Discussion

Oral cancer, like other types of head and neck malignancies, tends to metastasize to regional lymph nodes that are interconnected in a rich network [13]. Knowledge of lymphatic drainage patterns for oral cancers was first described by Rouviere in 1932 [14] and later modified into a simpler system (encompassing levels I to V) by Shah to recommend various lymphadenectomies based on metastatic risks [15]. Since then, further improvement has been made, and the system is now widely accepted for standardizing different neck dissection boundaries among practitioners [12, 16]. However, despite these revisions, some UMLNs have not yet been covered in traditional lymphatic levels, such as sublingual, buccinator and parotid lymph nodes. Although these UMLNs are not frequently encountered, they might sometimes reveal themselves during preoperative examinations or after failed treatment. Despite the tentative radiographic delineations by some authors [5], controversy still exists on whether the presence of UMLNs will affect the treatment or prognosis of SCCOC patients. Based on our statistics, we found that UMLNs may pose a serious threat to treatment success. First, contrary to our preconception, SCCOC patients with UMLNs in this cohort were generally those with locoregional failures, indicative of the occult or refractory nature of these special metastases. Second, most UMLNs could, as far as we were concerned, be regarded as indeterminate nodes for the middle locations between intraoral primary or recurrent lesions and cervical lymph nodes. These regions of treatment “vacuum” were often left “unfilled” or “unattended” despite vigorous standardized neck dissection attempts. This has been reflected and proven by the large proportion of isolated UMLNs in our series. In addition, the unfavorable outcomes of recurrent cases with UMLNs (RRG group) warned us about the importance of not only careful preoperative screening but also posttreatment monitoring of these possible UMLNs to avoid any delay of early detection.

Although generally described as the lymph nodes adjacent to the sublingual gland and underneath the floor of the mouth, the existence of sublingual lymph nodes has long been questioned [17]. The attention in such UMLNs was first raised for the coincidentally encountered lymph nodes during transoral resections of lesions in 1985. It was not until 2015 that the anatomic locations of sublingual lymph nodes were confirmed by Anarian in a cadaver study [18]. Although the presence was claimed to be approximately 30%, reports of metastases were largely inconsistent, as the incidence of these lesions varied from 8 to 17% [9, 10, 18]. However, when these UMLNs manifest during follow-up, the prognosis is adversely influenced [10, 18]. As shown in our study, when sublingual UMLNs occurred, the disease-specific survival rate was as low as 38.7%. If we narrowed the patients to those with recurrence (RRG group), the survival rate fell again to 9.1%. Some authors have claimed that the presence of such lymph nodes adds some clinical significance to the indications of more aggressive en bloc resection of primary oral cancers, especially those with advanced stages [7, 10, 19]. It is also widely acknowledged that for cases with advanced tongue lesions, sublingual gland and accompanying tissue will, to a large extent, not be preserved due to a margin safety concern [17]. Besides, discontinuous (separated) neck dissections may also lead to the possible residual diseases when the location of the primary lesions are in the vicinity of sublingual and buccal lymph nodes [6, 17]. However, for T1-2 or even some T3 cases with safe distances between primary lesions and the floor of the mouth, we recommend a thorough investigation for both physical and radiographic evidence. Additionally, we have noticed that the UMLN of those with tongue cancers were mostly in sublingual sites. These cases, however, were with a relatively poor survival outcome, which would be further complicated by adverse factors, such as recurrent or residual lesion status or unfavorable pathological grades. Therefore, from a surgical standpoint, we strongly advocate more aggressive in-continuity (en bloc) approaches when radiologically visible or palpable sublingual lymph nodes are found or any enlarged cervical lymph nodes are suspected especially for tongue primary with sublingual node.

Buccinator lymph nodes, also named facial lymph nodes, reside in/above the buccinator muscle and/or the fat of the buccinator space, with its border extending downwards to the lower mandible ridge [20]. These lymph nodes were rarely mentioned in studies focused on cervical metastases. Theoretically speaking, based on normal lymphatic drainages, tumors situated in the buccal mucosa, lower and upper gingiva, and palate are more likely to have buccinator lymph nodes as the upstream sentinel lymph nodes prior to any signs of cervical metastases [6, 20, 21]. However, despite the affluent lymphatic tissues in the buccal area, classic neck dissections (selective or radical) for oral cancer always exclude the removal of these buccinator lymph nodes [2, 12]. Such non-categorization in standard neck dissection procedures was claimed to be based on a low incidence of metastasis (9%) and possible coverage by prophylactic radiotherapy [6, 21]. As opposed to other studies, buccinator lymphatic metastases in our study were mostly encountered in patients for staged (secondary) neck dissections after previous resection of local malignancies [6, 22]. Thus, more concerns should be raised for suspected UMLNs in the buccal (supra-mandibular) intermediate region before these staged neck dissections, especially in SCCOC patients with upper gingival or palatal origins. Biopsies for buccinator nodes are also recommended for high-risk PG or SG patients with multiple cervical metastases or ENE features. On the other hand, buccinator UMLNs in patients with locoregional recurrence (RRG) were generally resistant to salvage treatment, according to the meagre survival (18.8%) of patients in our study. In addition, the recurrence of buccinator UMLNs after prior radiation therapies, from our perspective, might be partially attributable to possible reduced dosages around the mandible ridge for the prevention of osteonecrosis. However, such reduced dosages might in turn compromise the therapeutic effects, thereby resulting in these UMLNs.

According to the literature, most metastases in the parotid glands originate from cutaneous lesions in the scalps and ears, while metastases from SCCOCs are scarce, approximately only 2.9% [23–25]. From an anatomic standpoint, the chances are that SCCOCs may transport metastasis cells to superficial or deep parotid lymph nodes due to the retrograde drainage potential of intraoral mucosal lesions [8, 25]. This theoretical proposition was corroborated by our statistics, as most parotid UMLNs were from buccal or retromolar trigonal SCCOCs in proximity to parotid glands. Notably, only 3 patients in the PG group had parotid UMLNs. This indicated that when compared with sublingual and buccinator counterparts, parotid UMLNs were not among the first-echelon metastases for SCCOCs. Olsen also reported that parotid metastases were generally found in patients with recurrent instead of primary SCCOCs [25]. The low survival rate (15.4%) coincided with Harada’s statistics and implied that caution should be given to this group of patients regarding the decision for salvage surgery [8]. Alternatively, we contend that these patients may better be consulted for palliative or conservative regimens, especially when with additional disease burdens.

Apart from these specific UMLNs, other clinical variables were also considered to influence the survival outcomes. First, the outcomes may vary according to the various statuses of admission. We held the same notion that the prognoses in SCCOC patients with UMLN characteristics were greatly diversified—best in those for primary treatment and worst in those with recurrent lesions—irrespective of the UMLN subsites or sizes. Histories of preoperative irradiation or chemotherapy (*P* = 0.011) were found to be associated with worse survival outcomes in the whole case series. Despite no reports so far regarding the effects of preoperative adjuvant therapies in the control of UMLNs, we contended that such contradictory results were largely due to possible reduced dosages or neglected irradiated fields in these low-risk sites. Development of some UMLNs might have been avoided under the probable premises of enlarged field coverages or increased dosages. Besides, we are also the contender of en bloc resections for advanced primary SCCOC patients, especially those with deeper DOIs (*P* = 0.021), for the sake of resection radicality, as DOI has been recognized as significant adverse prognosticator for overall survival [26, 27]. Though with significant results in this study, PNI, as a core histopathological feature of SCCOCs, is also indicative of the disease aggressiveness [26, 28]. The result of PNI, as we perceived, might be caused by the small sample size of this study. In addition, we found that the factor, ENE in all UMLNs (*P* = 0.025) was significantly related to poor survival, which was also consistent with the results in other investigations [29–31]. The types of neck dissections, implying the extent of metastases, also affected the survival of UMLN patients [32]. In addition, we found that the primary or recurrent sites (*P* = 0.035), especially those with lesions in retromolar trigones or mouth floors, shared the most undesirable outcomes. Surprisingly, we found no significant effects in postoperative adjuvant therapies for patients with UMLNs, despite the general trend towards disease control improvement in others with metastatic SCCOCs [1, 3, 25].

### Limitations of the study

Though this study had multi-institutional cooperation, on account of the rarity of UMLNs, the sample size was still relatively small. In addition, errors of selection and treatment bias were inevitable due to a retrospective design.

## Conclusions

Early surgical interventions are warranted in patients with sublingual or buccinator metastases, while caution should be given to those with parotid metastases. Aggressive en bloc (in-continuity) resections may be mandatory in advanced oral cancer cases for close anatomic locations with possible buccal or sublingual metastases. For cases with primary advanced tongue cancers, sublingual nodes should be removed due to a possible concern for middle-zone residual lesions. Palliative rather than aggressive retreatment should also be given to those with parotid metastases, due to poorer survival outcomes. Overall, although scarce, awareness of these potential UMLNs should be emphasized to all practitioners dealing with SCCOCs in both pretreatment evaluations and posttreatment follow-up.

## Supplementary Information

**Additional file 1: Supplementary figure 1**. The Kaplan-Meier curves of the significant variables in Cox analyses. A. Type of neck dissection; B. Primary or recurrent site; C. UMLN subsites.

**Additional file 2: Supplementary figure 2**. The Kaplan-Meier survival curves of different admission groups.

**Additional file 3: Supplementary figure 3**. Recurrent SCCOC case with a buccinator lymph node metastasis. A: Preoperative view of the patient. B: The intraoperative exposure of the metastatic buccinator lymph nodes.

**Additional file 4: Supplementary figure 4**. Primary SCCOC case with a sublingual lymph node metastasis. A: The intraoperative exposure of metastatic sublingual lymph node. B: The excised sublingual lymph nodes with metastatic changes in section.

**Additional file 5: Supplementary figure 5**. A: The intraoperative exposure of metastatic sublingual lymph node. B: The excised surface of metastatic sublingual lymph node.

**Additional file 6: Supplementary figure 6.** The intraoperative exposure of metastatic sublingual lymph node (next to inner surface of mandible).

**Additional file 7: Supplementary figure 7**. Recurrent SCCOC case with ipsilateral parotid lymph nodes metastasis. A: The axial CT view of the recurrent cancer in left floor of mouth area (the direction of the arrow). B: The axial CT view of the metastatic left parotid lymph nodes of this patient (the direction of the arrow).

**Additional file 8: Supplementary figure 8**. The patient had received buccal carcinoma resection and ipsilateral radical neck dissection ten months ago. And he was treated with radiation after the surgery. Two months ago, he was found ipsilateral isolated parotid metastasis at the follow-up. A: Preoperative view of the patient, the an arrow points to the parotid lymph node metastasis/ B: The axial PET-CT view of the metastatic lymph node (the direction of the arrow). C\D: The coronal PET-CT view of the metastatic parotid lymph node(the direction of the arrow).

**Additional file 9: Supplementary figure 9**. A: The intraoperative exposure of metastatic parotid lymph node. B: The range of the tumor resection. Part of the ascending ramus and angle of the mandible were resected.

**Additional file 10: Supplemental table 1**. The Prognostic Factors For The Suspected Residual Group.

**Additional file 11: Supplemental table 2**. The Prognostic Factors For Tongue Cancer Patients With Sublingual Node Metastases.

## Data Availability

The data that support the findings of this study are available from the corresponding author upon reasonable request

## Abbreviations

SCCOC: Squamous cell carcinoma of oral cavity
UMLN: Unconventional metastatic lymph nodes
NCCN: National Comprehensive Cancer Network
SCC: Squamous cell carcinomas
AJCC: American Joint Committee on Cancer
DOI: Depth of invasion
ENE: Extranodal extensions
ISND: Ipsilateral selective neck dissection (levels I to III)
IRND: Ipsilateral radical neck dissection (levels I to V)
BND: Bilateral neck dissection
CND: Contralateral neck dissection
EGFR: Anti-epithelial growth factor receptor
PNI: Perineural invasion
