# Supplementary figures and images for: Regional metastasis to anatomies beyond traditional neck dissection boundaries: a multi-institutional analysis focused on unconventional metastases in oral cancer patients
Source: World J Surg Oncol. 2020 Oct 28;18:281. doi: 10.1186/s12957-020-02057-6 (PMC7594434; doi:10.1186/s12957-020-02057-6)

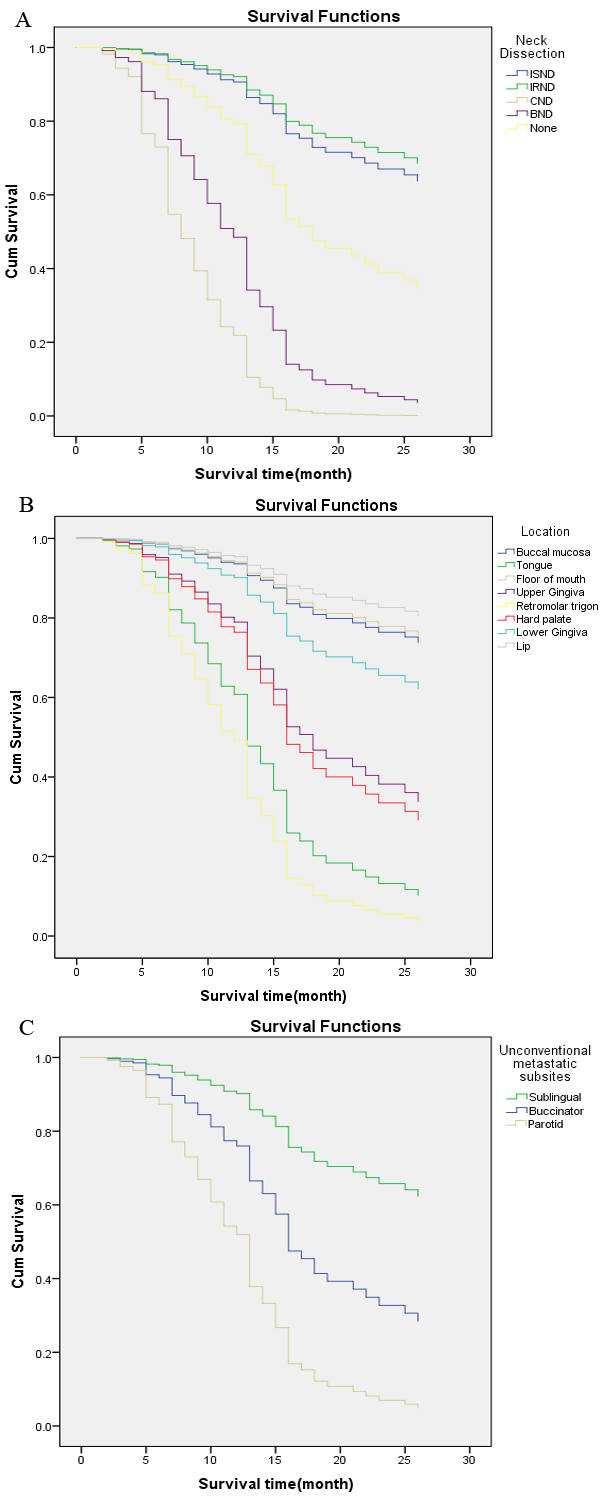

Supplement: Supplementary file 1 — Additional file 1: Supplementary figure 1. The Kaplan-Meier curves of the significant variables in Cox analyses. A. Type of neck dissection; B. Primary or recurrent site; C. UMLN subsites. [file 12957_2020_2057_MOESM1_ESM.tif]

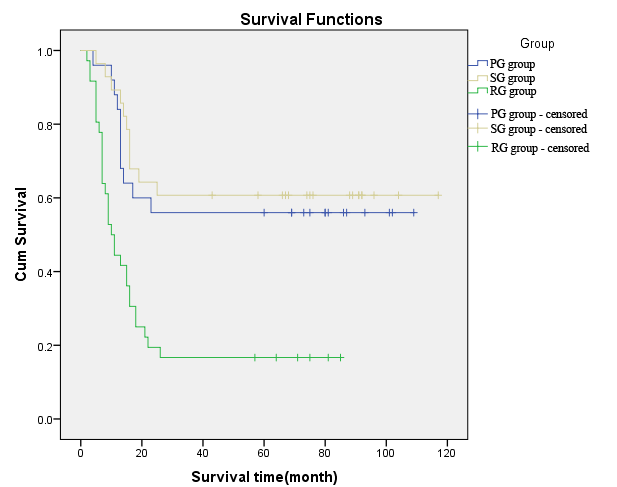

Supplement: Supplementary file 2 — Additional file 2: Supplementary figure 2. The Kaplan-Meier survival curves of different admission groups. [file 12957_2020_2057_MOESM2_ESM.tif]

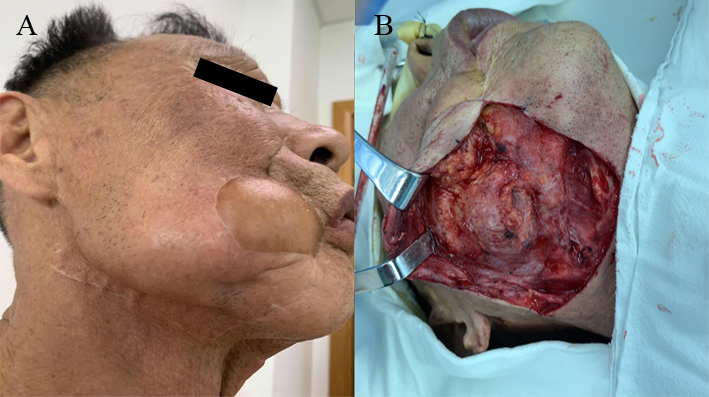

Supplement: Supplementary file 3 — Additional file 3: Supplementary figure 3. Recurrent SCCOC case with a buccinator lymph node metastasis. A: Preoperative view of the patient. B: The intraoperative exposure of the metastatic buccinator lymph nodes. [file 12957_2020_2057_MOESM3_ESM.tif]

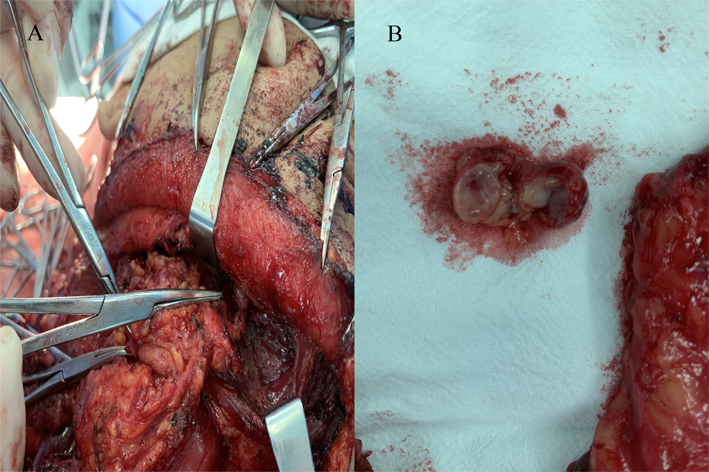

Supplement: Supplementary file 4 — Additional file 4: Supplementary figure 4. Primary SCCOC case with a sublingual lymph node metastasis. A: The intraoperative exposure of metastatic sublingual lymph node. B: The excised sublingual lymph nodes with metastatic changes in section. [file 12957_2020_2057_MOESM4_ESM.tif]

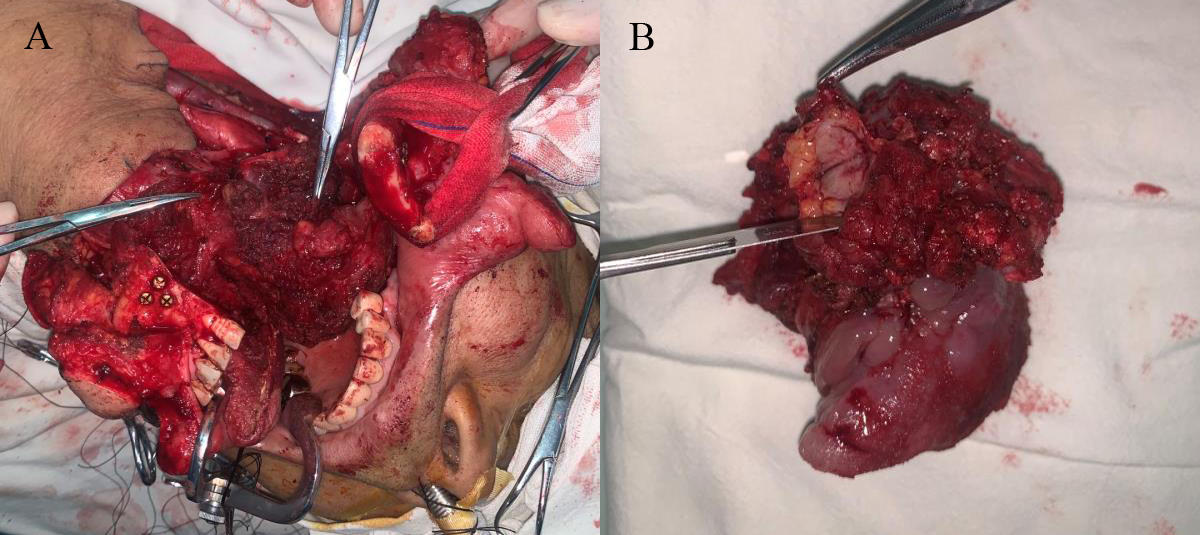

Supplement: Supplementary file 5 — Additional file 5: Supplementary figure 5. A: The intraoperative exposure of metastatic sublingual lymph node. B: The excised surface of metastatic sublingual lymph node. [file 12957_2020_2057_MOESM5_ESM.tif]

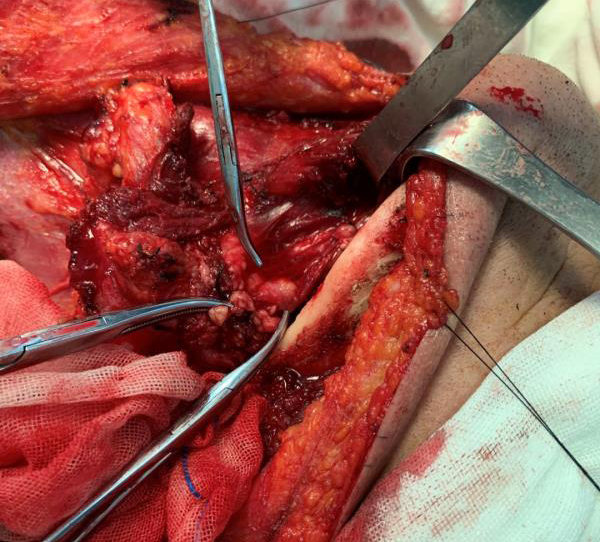

Supplement: Supplementary file 6 — Additional file 6: Supplementary figure 6. The intraoperative exposure of metastatic sublingual lymph node (next to inner surface of mandible). [file 12957_2020_2057_MOESM6_ESM.tif]

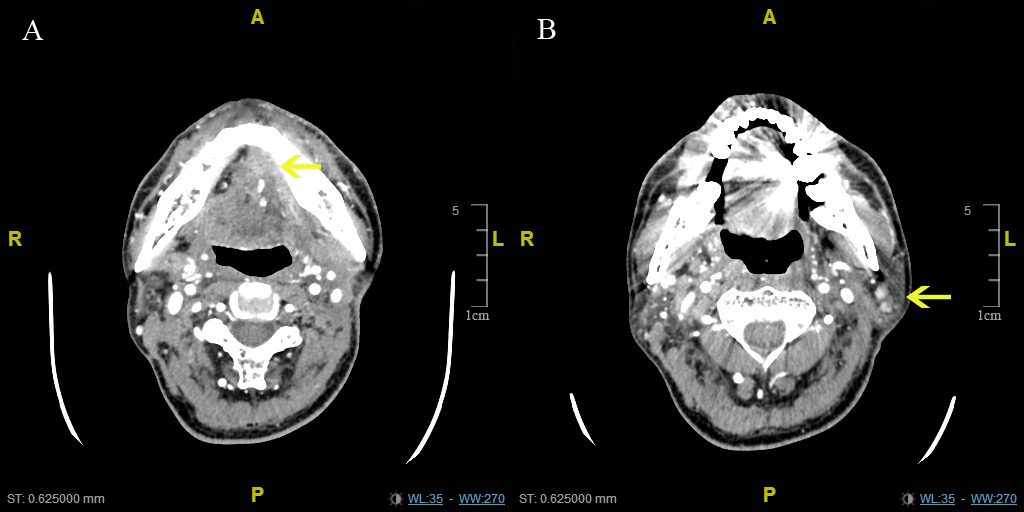

Supplement: Supplementary file 7 — Additional file 7: Supplementary figure 7. Recurrent SCCOC case with ipsilateral parotid lymph nodes metastasis. A: The axial CT view of the recurrent cancer in left floor of mouth area (the direction of the arrow). B: The axial CT view of the metastatic left parotid lymph nodes of this patient (the direction of the arrow). [file 12957_2020_2057_MOESM7_ESM.tif]

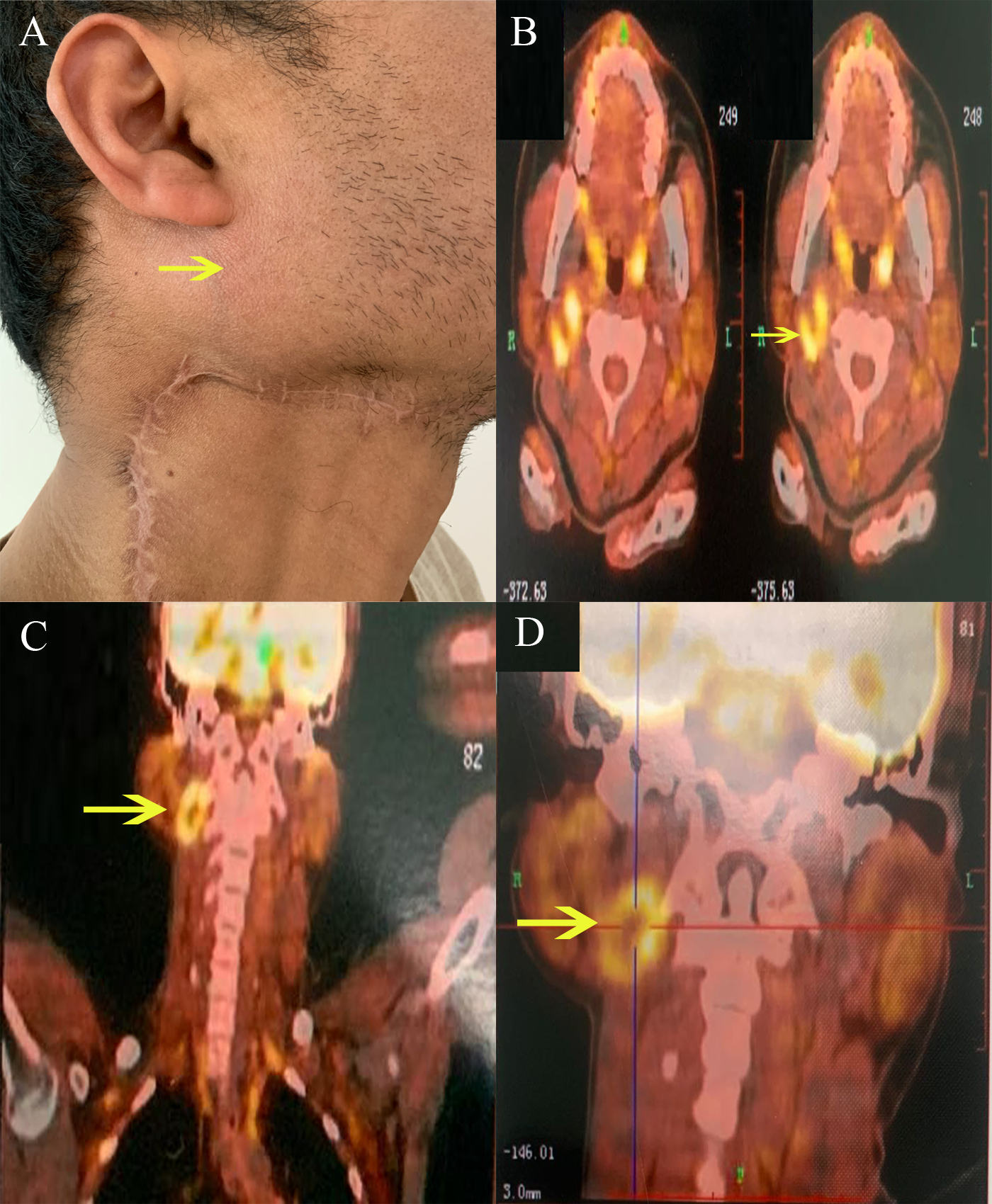

Supplement: Supplementary file 8 — Additional file 8: Supplementary figure 8. The patient had received buccal carcinoma resection and ipsilateral radical neck dissection ten months ago. And he was treated with radiation after the surgery. Two months ago, he was found ipsilateral isolated parotid metastasis at the follow-up. A: Preoperative view of the patient, the an arrow points to the parotid lymph node metastasis/ B: The axial PET-CT view of the metastatic lymph node (the direction of the arrow). C\D: The coronal PET-CT view of the metastatic parotid lymph node(the direction of the arrow). [file 12957_2020_2057_MOESM8_ESM.tif]

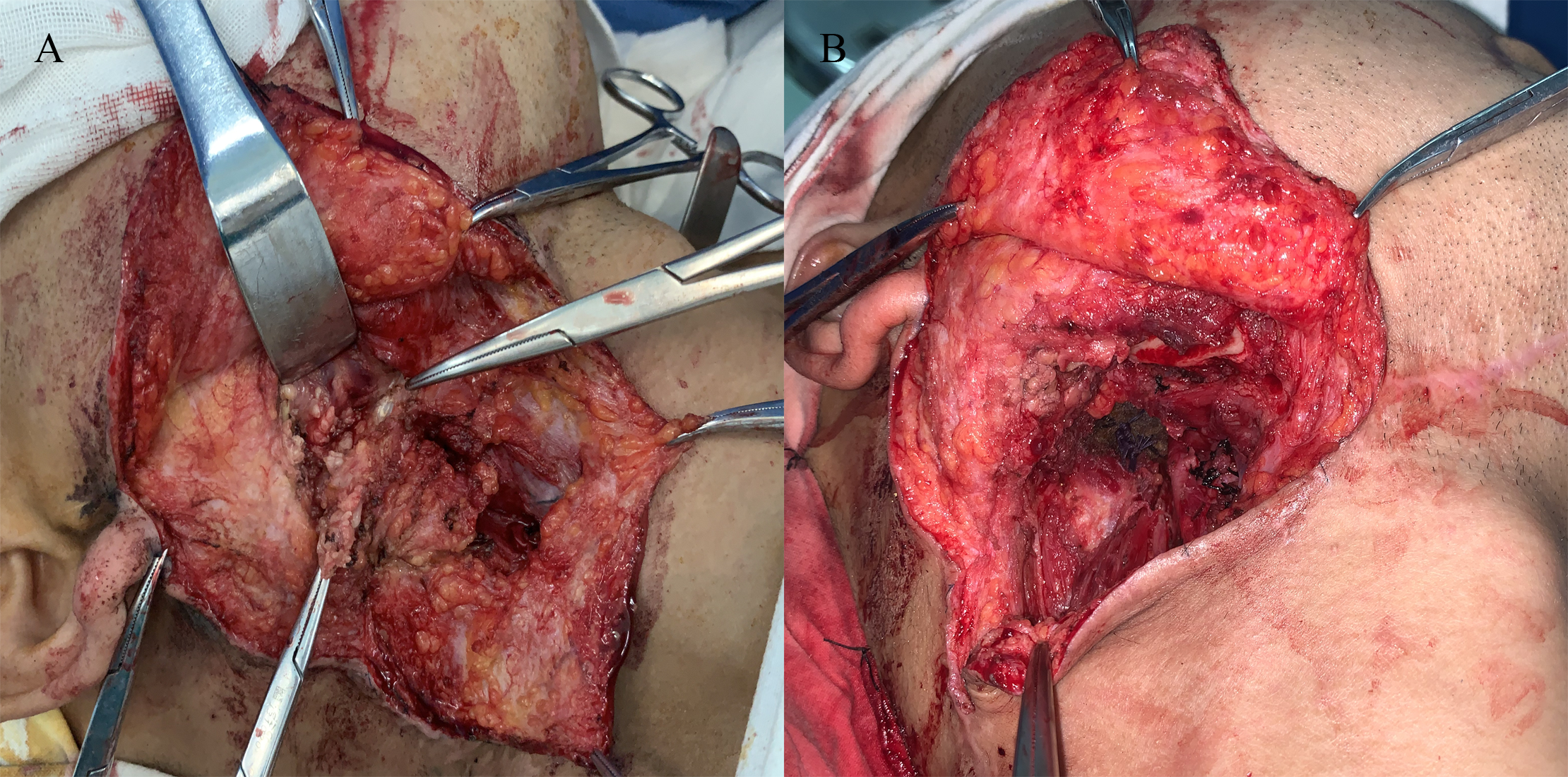

Supplement: Supplementary file 9 — Additional file 9: Supplementary figure 9. A: The intraoperative exposure of metastatic parotid lymph node. B: The range of the tumor resection. Part of the ascending ramus and angle of the mandible were resected. [file 12957_2020_2057_MOESM9_ESM.tif]
