# Supplementary material for: Regional metastasis to anatomies beyond traditional neck dissection boundaries: a multi-institutional analysis focused on unconventional metastases in oral cancer patients
Source: World J Surg Oncol. 2020 Oct 28;18:281. doi: 10.1186/s12957-020-02057-6 (PMC7594434; doi:10.1186/s12957-020-02057-6)
Supplement: Supplementary file 10 — Additional file 10: Supplemental table 1. The Prognostic Factors For The Suspected Residual Group. [file 12957_2020_2057_MOESM10_ESM.docx]

| **Supplemental table 1. The Prognostic Factors For The Suspected Residual Group** | | | |
| --- | --- | --- | --- |
| Variables (n) | The overall survival rate | p Value | |
|  |  | The Kaplan–Meier Method | Cox Model |
| Age |  | 0.398 | NA |
| 33-59(23) | 39.1% |  |  |
| 60-87(13) | 30.8% |  |  |
| Gender |  | 0.721 | NA |
| Male(20) | 45.0% |  |  |
| Female(16) | 25.0% |  |  |
| Histories of smoking or alcohol |  | 0.165 | NA |
| Yes(16) | 50.0% |  |  |
| No(20) | 25.0% |  |  |
| Premalignant mucosal diseases |  | 0.296 | NA |
| Yes(10) | 40.0% |  |  |
| No(26) | 34.6% |  |  |
| Pathological grade |  | 0.035 | NA |
| I(1) | 100.0% |  |  |
| II(24) | 45.8% |  |  |
| III(11) | 9.1% |  |  |
| Treatment status |  | 0.117 | NA |
| Primary lesions for treatment (PG)(12) | 50.00% |  |  |
| Staged (secondary) neck dissections (SG)(9) | 44.40% |  |  |
| Recurrent or residual lesions for salvage surgery (RRG)(15) | 20.00% |  |  |
| Unconventional metastatic subsites |  | 0.576 | NA |
| Buccinator(5) | 20.00% |  |  |
| Sublingual(25) | 40.00% |  |  |
| Parotid*(6) | 33.30% |  |  |
| Number of metastatic lymph nodes in sublingual |  | 0.095 | NA |
| 1(31) | 38.7% |  |  |
| 2(5) | 20.0% |  |  |
| Lymph nodes size of sublingual node |  | 0.001 | NA |
| 0-3cm(26) | 46.2% |  |  |
| >3cm(10) | 10.0% |  |  |
| Extranodal extention (ENE) in sublingual node |  | 0.001 | 0.003 |
| Yes(11) | 9.1% |  |  |
| No(25) | 48.0% |  |  |
| Contralateral metastasis |  | 0.002 | 0.008 |
| Yes(12) | 8.3% |  |  |
| No(24) | 50.0% |  |  |
| Cervical lymph node metastasis |  | 0.005 | NA |
| Yes(21) | 9.5% |  |  |
| No(15) | 73.3% |  |  |
| Extranodal extention (ENE) in cervical lymph nodes |  | 0.001 | NA |
| Yes(8) | None |  |  |
| No(28) | 46.4% |  |  |
| Postoperative adjuvant therapies |  | 0.439 | NA |
| Radiotherapy(14) | 35.7% |  |  |
| Chemotherapy(1) | None |  |  |
| Radio-chemotherapies(10) | 20.0% |  |  |
| None(11) | 54.5% |  |  |
|  |  |  |  |
| NA：Not significant. |  |  |  |
| *:Including a case which is with both the buccinator and the parotid metastases, but mostly in the parotid. | | |  |
