# Supplementary material for: Regional metastasis to anatomies beyond traditional neck dissection boundaries: a multi-institutional analysis focused on unconventional metastases in oral cancer patients
Source: World J Surg Oncol. 2020 Oct 28;18:281. doi: 10.1186/s12957-020-02057-6 (PMC7594434; doi:10.1186/s12957-020-02057-6)
Supplement: Supplementary file 11 — Additional file 11: Supplemental table 2. The Prognostic Factors For Tongue Cancer Patients With Sublingual Node Metastases. [file 12957_2020_2057_MOESM11_ESM.docx]

| **Supplemental table 2. The Prognostic Factors For Tongue Cancer Patients With Sublingual Node Metastases** | | | |
| --- | --- | --- | --- |
| Variables (n) | The overall survival rate | p Value | |
|  |  | Kaplan–Meier Method | Cox Model |
| Age |  | 0.109 | NA |
| 33-59(14) | 35.7% |  |  |
| 60-87(8) | 50.0% |  |  |
| Gender |  | 0.211 | NA |
| Male(10) | 60.0% |  |  |
| Female(12) | 25.0% |  |  |
| Histories of smoking or alcohol |  | 0.138 | NA |
| Yes(9) | 66.7% |  |  |
| No(13) | 23.1% |  |  |
| Premalignant mucosal diseases |  | 0.509 | NA |
| Yes(8) | 37.5% |  |  |
| No(14) | 42.9% |  |  |
| Treatment status |  | 0.001 | 0.003 |
| Primary lesions for treatment (PG)(10) | 60.00% |  |  |
| Staged (secondary) neck dissections (SG)（6) | 50.00% |  |  |
| Recurrent or residual lesions for salvage surgery (RRG)(6) | None |  |  |
| Pathological grade |  | 0.003 | 0.001 |
| I(1) | 100.0% |  |  |
| II(18) | 44.4% |  |  |
| III(3) | None |  |  |
| Number of metastatic lymph nodes in sublingual |  | 0.117 | NA |
| 1(21) | 42.9% |  |  |
| 2(1) | None |  |  |
| Lymph nodes size of sublingual node |  | 0.023 | NA |
| 0-3cm(20) | 45.0% |  |  |
| >3cm(2) | None |  |  |
| Extranodal extention (ENE) in sublingual node |  | 0.008 | NA |
| Yes(4) | None |  |  |
| No(18) | 50.0% |  |  |
| Contralateral metastasis |  | 0.003 | NA |
| Yes(8) | 12.5% |  |  |
| No(14) | 57.1% |  |  |
| Cervical lymph node metastasis |  | 0.002 | NA |
| Yes(15) | 13.3% |  |  |
| No(7) | 100.0% |  |  |
| Extranodal extention (ENE) in cervical lymph nodes |  | 0.028 | NA |
| Yes(3) | None |  |  |
| No(19) | 47.4% |  |  |
| Postoperative adjuvant therapies |  | 0.208 | NA |
| Radiotherapy(11) | 27.3% |  |  |
| Radio-chemotherapies(6) | 33.3% |  |  |
| None(5) | 80.0% |  |  |
|  |  |  |  |
| NA：Not significant. |  |  |  |
